# Mobility and Muscle Strength Together are More Strongly Correlated with Falls in Suburb-Dwelling Older Chinese

**DOI:** 10.1038/srep25420

**Published:** 2016-05-05

**Authors:** Xiuyang Wang, Yixuan Ma, Jiazhong Wang, Peipei Han, Renwei Dong, Li Kang, Wen Zhang, Suxing Shen, Jing Wang, Dongfang Li, Maoran Zhou, Liancheng Wang, Kaijun Niu, Qi Guo

**Affiliations:** 1 Department of Rehabilitation Medicine, TEDA International Cardiovascular Hospital, Cardiovascular Clinical College of Tianjin Medical University, Tianjin, China; 2 Department of Rehabilitation and Sports Medicine, Tianjin Medical University, Tianjin, China; 3 Department of Rehabilitation Medicine, Tianjin Hospital, Tianjin, China; 4 Nutritional Epidemiology Institute, Tianjin Medical University, Tianjin, China; 5 School of Public Health, Tianjin Medical University, Tianjin, China

## Abstract

Falls are common in older adults and result in adverse outcomes. Impaired mobility and poor muscle strength have been consistently identified as the main contributors to falls. We choose three easy-to-perform tests (i.e. Timed Up and Go test (TUGT), walking speed (WS) and grip strength (GS)) in order to assess mobility and muscle strength to further define their relationship with falls. This study is cross-sectional, consisting of 1092 residents over 60-year-old; 589 were female. 204 (18.68%) participants reported falling at least once in the past year. It was found that, of the three tests evaluated independently, a TUGT < 9.1750 s had the strongest association with fewer falls. When evaluating these tests as pairs, the combination of a TUGT < 9.1750 s and a WS < 0.9963 m/s was the best protective indicator of falls after adjusting for age, sex and other variables. When evaluating all three tests in conjunction with each other, the combination of a TUGT < 9.1750 s, a WS < 0.9963 m/s, and a GS > 0.3816 was most correlated with less possibility of falls. The combination of a better TUGT performance, a stronger GS, and a slower WS is the most strongly correlated with less possibility of falls.

One-third of adults living independently in residential community older than 65 years falls each year^1^. Falls result in major adverse outcomes in older adults including injury, institutionalization, and death^2^,^3^. Because of the high rate of falls in older adults and the severe consequences of falls, it is very important to find the factors correlated with falls in older adults to help us achieve better understanding, develop a screening tool and develop prevention strategies for falls. Although various factors are correlated with falls, impaired balance and mobility have been consistently identified as the main factors^4^.

The Timed Up and Go test (TUGT) require the individual to stand up from a chair, walk 3 m, turn around, walk back to the chair and sit down again. Walking speed (WS) require participants to stand with both feet touching the starting line and to begin walking 4 m at their usual pace after a verbal command. Both TUGT and WS are two simple clinical tests usually used to measure balance and mobility. Muscle strength is also an important factor for balance and mobility ability^5^,^6^,^7^. The TUGT is commonly used as a screening tool for fall risk in community-dwelling older adults. The longer time the TUGT takes, the lower the mobility of the participant, and the higher the risk of falls^8^,^9^. WS is another physical index that has been found to be correlated with the risk of falls in previous studies. Many studies found quicker WS represented better mobility^10^,^11^ and some studies even found it is better to assess functional skills like walking speed rather than the TUGT^12^. Muscle strength also plays an important role in fall prevention. Grip strength (GS) is an indicator of trunk muscle strength and lower limb muscle strength^13^,^14^, and can also be used to identify the risk of falling among the elderly^15^.

Although previous studies have proved these three tests may be correlated with fall risk, it hard to identify a single test that can predict the multiple factors involved in falls, and there are also studies that suggest a single tool does not have sufficient power to predict falls in independently living senior adults^8^,^16^. Specific diseases and impairments may also affect specific aspects of function, which may then determine the characteristics of an individual’s likelihood of falling.

Measuring with different types of measurement increases reliability^17^, thus measurement accuracy may be gained by using multiple methods to measure fall risk. However, some research found quicker WS with good muscle strength may lead to more falls than normal^18^,^19^, which indicates that the role WS plays could change depending on what combination of tests are used. Therefore, this study investigated suburb-dwelling older adults age ≥60 years in Hangu, Tianjin, China. In China, more than 70% of the older adults live in suburban areas. Older individuals have a relatively low level of medical treatment and display poor health^20^, which is why particular attention should be given to those living in the suburbs^21^. The purpose of this study is to discover whether the TUGT, WS, GS, or some combination of the aforementioned tests has the strongest association with falls in suburban community-dwelling older adults.

## Materials and Method

### Ethics statement

This research was approved by the Ethics Committee at Tianjin Medical University, and the methods were carried out in accordance with the principles of the Declaration of Helsinki. All records were anonymized and no individual information can be identified.

### Subject

Our study population consisted of residents of the Hangu area of Tianjin, China who were adults aged ≥60 years old and who had joined China’s national free physical examination program. All potential subjects were invited to participate in a comprehensive geriatric assessment. Individuals who needed assistance with their daily activities were included as long as they were able to finish the study’s tasks without assistance. If these individuals required assistance to complete the tasks, they were excluded. Cancer patients were also excluded because cancer’s progressive nature can markedly affect physical performance. Individuals with visual impairments (i.e. glaucoma, cataracts, and myopia and presbyopia that were not adequately corrected) who reported their impairments interfered with their daily activities were also excluded. Individuals taking medications that can cause falls (i.e. psychotropic drugs, cardiovascular drugs, hypoglycemic agents, non-steroidal anti-inflammatory drugs, analgesics, dopaminergic drugs, Parkinson’s disease drugs, or more than four kinds of complex drugs) in the past year were also excluded. Of those invited, 1181 agreed to participate in the survey and gave their informed written consent. 89 individuals were excluded, and the final study population consisted of 1092 subjects (average age: 67.35 ± 5.93 y; 44.23% male).

### Definition of Fall

In this study, a fall is defined as any event that results in a bodily change that forces an individual to inadvertently land on the ground or a lower level; the fall cannot be caused by a violent blow, loss of consciousness, sudden onset paralysis, or epileptic seizure. Participants reported falls by themselves and who reported at least one fall in the past 1 year were categorized as “fallers”.

### Performance-based assessment

The performance-based assessments consisted of three physical tests: the TUGT, a test evaluating WS, and a test evaluating GS. All tests were conducted by postgraduate students in the healthcare field who received special training to perform these assessments. The TUGT assesses the number of seconds needed for an individual to stand up from a chair, walk 3 m at his usual pace past a line on the floor, turn around, walk back to the chair, and sit down again with his back against the chair. WS was assessed with the 4-m walk test; participants were instructed to stand with both feet touching the starting line and to begin walking at their usual pace after a verbal command. The time between activation of the first and second photocells was recorded. The shorter time of two trials was used for analyses. The participants were allowed to use a gait-assistance device. Grip strength (kg) was used as a measure of muscle strength and was quantified using a handheld dynamometer (GRIP-D; Takei Ltd, Niigata, Japan). Participants were asked to exert their maximum effort twice using their dominant hand; the stronger grip strength was used for analyses. In this study, hand grip strength was adjusted in accordance with the participant’s body weight (GS = grip strength (kg)/body weight (kg)). Grip strength is correlated with the muscle strength of the lower limbs^14^ and body size^22^, while leg strength/body weight (weight bearing index, WBI) is predictive of functional ability in older adults^23^, therefore, we used body weight to adjust grip strength in order to ensure we were evaluating muscle strength independent of body size. This method of standardization has been previously recommended in order to normalize and improve physical function testing results^24^,^25^.

### Assessment of other variables

All the participants were invited to a face-to-face interview to answer a standardized questionnaire after they completed their medical examination. The questionnaire included questions about age, sex, marital status, smoking habits (current smoker or not), and drinking habits (drinking alcohol once a week, drinking in the past, and never drinking were all considered as no drinking). Height and weight were recorded using a standard protocol. Body mass index was calculated as weight in kilograms divided by height in meters squared. Participants self-reported their past medical history (e.g. diabetes). The prevalence of specific medical conditions was established using standardized criteria that combined information from participants’ self-reported medical history, physicians’ diagnoses, and current and past medications and therapies. The subjects did not receive a physical exam. As described in previous literature, physical activity was assessed using the short form of the International Physical Activity Questionnaire (IPAQ) in the Chinese language^26^. We used the Geriatric Depression Scale (GDS) to evaluate the degree of depression of participants; GDS scores of 0–10 were classified as having no depression, scores of 11–20 were classified as slight depression, and scores of 21–30 were classified as moderate to severe depression. An Electrical Bioimpedance Measurement (Inbody 720, Biospace Ltd, Korea) was used to analyze participants’ body compositions including muscle mass. Quantitative ultrasound (OsteoPro UBD2002A, BMTECH. WorldWide Co., Ltd, Korea) was used to measure bone mineral density. T > −1 was defined as normal bone mineral density, −2.51 < T ≤ −1 was defined as osteopenia, and T ≤ −2.5 was defined as osteoporosis.

### Statistics

SPSS17.0 software for Windows was used for statistical analyses. Hand grip strength was standardized by weight. Chi-square tests were used for categorical variables, and ANVOA analyses were used for continuous variables to compare the faller and non-faller groups. The receiver operating characteristic curve (ROC) was used to determine the cutoff points for falls based on the TUGT, WS, and GS. Binary logistic regression analyses were used to assess the associations and interactions between a history of falling, TUGT, WS, and GS. Only age and sex were included in the logistic regression analysis in model 1, while in model 2, all variables that had significant relationships with falls were included in the logistic regression analysis. Significance was set at two-tailed P < 0.05 for Chi-square tests, ANVOA analyses and logistic regression analysis.

## Result

### Comparison of characteristics according to falls in past 12 months

There were 1092 adults (589 females and 503 males), all of whom were above 60 years of age, in this study. 204 of all participants reported falling at least once in the past year. Tables 1 and 2 summarize the statistically significant characteristics of the faller and non-faller groups. The average age of the faller group is significantly older than the non-faller group. Older individuals have more falls (15.6% of 60–64.9 year-old, 17.2% of 65–69.9 year-old, 25.9% of 70–74.9 year-old and 26.4% older than 75 years fell in the past year). The faller group had significantly more females, more subjects with depression, and more subjects with diabetes than the non-faller group. The faller group was less likely to be living with a spouse than the non-faller group. The faller group had less muscle mass than the non-faller group. Regarding physical performance, the non-faller group took shorter time for TUGT, had stronger GS and slower walking speed than the faller group. All of the variables shown in Tables 1 and 2 are included in model 2.

### The cutoff point of TUGT, GS and WS according to ROC curve

According to the receiver operating characteristic curve (ROC), the cutoff point of TUGT for fall is 9.7150 s, and the Area Under the Curve (AUC) is 0.609. The cutoff point of TUGT for falls increases with age: 9.05 seconds for 60–64.9 years old, 9.77 seconds for 65–69.9 years old, 9.96 seconds for 70–74.9 years old, and 10.86 seconds for 75 years or older. The cutoff point of GS for falls is 0.3816, and the AUC is 0.594. The cutoff point of WS for falls is 0.9963 m/s, and the AUC is 0.586 (Table 3). According to the cutoff points, we defined good performance on the tests as “+”, and bad performance as “−” (TUGT+: TUGT < 9.7150 s; TUGT−: TUGT > 9.7150 s; GS+: GS > 0.3816; GS−: GS < 0.3816; WS+: WS > 0.9973 m/s; WS−: WS < 0.9963 m/s).

### Logistic regression analysis of the relationship between falls and TUGT, WS, GS and their combination

Variables shown in Table 1 are all included in model 2. When evaluating these tests independently, TUGT+ had the strongest association with less possibility of falls of the three physical tests results (odd ratio (OR) values of crude, model 1 and model 2 are 0.534, 0.609 and 0.622 respectively) (Table 4). When evaluating these tests as pairs, the combination of TUGT+ and GS+ has the strongest relationship with fewer falls (OR = 0.390), while in model 1 and model 2, the combination of TUGT+ and WS− has the strongest relationship with fewer falls (OR values are 0.510 and 0.555 respectively) (Table 4). When compared to the results of each test evaluated independently and in pairs, the combination of all three test results had the strongest relationship with a lower possibility of falls when TUGT+, GS+ and WS− (OR values are 0.291, 0.398 and 0.394 respectively) (Table 4).

## Discussion

In our study, we examined three tests commonly used to predict falls, including the TUGT, GS and WS. The study indicated that the combination of TUGT+, GS+ and WS− is most closely correlated with fewer falls. When using only two tests, the combination of TUGT+ and WS− is most often correlated with fewer falls. When comparing three test results independently, TUGT+ was the best protective indicator of falls.

According to our study, the prevalence of falls during a single year was 18.68%, which was similar to the result in previous studies among mainly Chinese populations (between 16.3–21.3%)^27^,^28^,^29^. The prevalence of falls in female was 22.2%, and the prevalence of falls in male was 14.3%. Older age is positively correlated to falls. The rate was lower than the reported annual falls (35–40%) in community-dwelling older people in western countries^30^. Although the age and gender composition between the two groups are similar, culture, lifestyle, daily life activities, family structure and the social system may be responsibility for the difference, perhaps because older people in western countries are more independent in daily life and outdoor activities.

The TUGT has been widely adopted in both hospitalized and community-dwelling populations to measure balance and screening fall risk in older adults according to the literatures^9^,^31^,^32^. In our study, the TUGT has the best AUC for fall among the three physical tests-TUGT, GS and WS, and continued to have the strongest association with falls after adjusting for age, sex and other variables. As a short and simple clinical balance test, the success of TUGT in predicting falls is likely related to the sequencing of several important mobility skills, such as turning and sit-to-stand transitions that require balance control, as well as straight-ahead gait^33^. However, the TUGT suffers from the same limitations as the other functional clinical scales, since it is not possible to separate which balance and gait subcomponents are affected^34^. A recent systematic review and meta-analysis indicated that the TUGT is more useful at ruling out the possibility of falls rather than positively identifying individuals classified as high risk, thus demonstrating that the diagnostic accuracy of the TUGT test is limited. The literature also suggests TUGT should not be used in isolation to identify individuals at high risk of falls^8^.

The cutoff point of TUGT for falls is 9.7150 seconds in our study, while another Chinese population based study in Hong Kong found older adults who take longer than 14 seconds to complete the TUGT have a high risk for falls^35^. This difference may be caused by age. In our study, the average age of individuals was 67.35 ± 5.93 years while in of the Hong Kong study, the average age of individuals was 73.17 ± 6.26 years. Older age participants could potentially take a longer time to complete TUGT than those of younger age. Additionally, our participants were more active and independent while participants in that Hong Kong study were ambulatory cared.

Hand grip strength is an estimation of isometric strength in the upper extremity, but also correlates with strength in other muscle groups, and therefore, is considered an estimate of the overall strength^22^. Greater muscle strength can provide more stable support during walking, and previous studies show that reduced muscle strength plays an important role in falls in older adults^13^,^14^. Furthermore, exercises which improve muscle strength will help to reduce the fear of falls, and may have positive outcome in reducing falls in future^36^. But in our study, after adjusting for age, sex and other variables, data analysis shows that GS and falls are weakly correlated. Other randomized controlled studies also have reached the same result^37^,^38^. In those studies, although muscle strength increased in an exercise group, it did not result in a significant difference between the exercise group and control group in falls during the follow-up period. This may explained by the complex causes of falls. A systematic review of resistance training also suggested that muscle strengthening alone might not improve balance^39^.

In this cross-sectional study, data shows WS has no relationship with falls in older adults after adjusting for other variables. Some previous studies found slower WS could lead to an increase of falls^6^,^11^,^40^. A 20-month cohort study found that each 10 cm/s decrease in gait speed was correlated with a 7% increased risk for falls, and the cutoff point of walking speed was 0.7 m/s^11^. Our participants are comparatively younger than those of that study (67.4 years vs. 79.6 years) and the cutoff point of WS we record was 0.9963 m/s.

The fact that WS and falls are independent of one another in our study may be explained by a previous study which found there is a non-linear relationship between WS and falls. This study indicated a greater risk of outdoor falls in faster walkers and greater risk of indoor falls in slower walkers^18^. In addition, our data reaches similar conclusions. When we combined the TUGT and WS tests to study fall risk, the non-faller group shows slower WS than the faller group, which may be because the TUGT performance signifies good mobility and WS is part of the TUGT. Thus, walking speed will vary with change in mobility. For example, in older adults who have good mobility, a person who walks more carefully and slowly may have fewer chances to fall than one who is rushed. Pavol *et al.*^19^ has confirmed this in his study, which indicated individuals who had more muscle strength and a higher walking velocity had a higher risk of falling. He believes stronger muscle strength and a higher walking velocity would cause a during-step or elevating-response fall rather than an after-step fall. Given all this, one must take other mobility tests into account when using WS to study falls.

The mechanisms underlying falls are complex; for example, age, body shape, vestibular sense, proprioception, muscle strength, vision, drug use, oral health, and environment are all contributors. Similar to our result, many literature have found older age leads to more falls^41^,^42^. Body shape also plays an important role in falls, as Mitchell *et al.* found obese individuals (BMI ≥ 30 kg/m^2^) have a 31% higher fall risk than healthy weight participants (18.5 kg/m^2^ ≤ BMI ≤ 24.9 kg/m^2^)^43^. Clear and precise vision provides the necessary information to navigate effectively through the world and to control the movement of the body, which in turn helps control upright posture. Input from the eyes is used by the central nervous system (CNS) to create a spatial map of the environment, through which we can quickly assess the speed and direction of moving objects and locate hazards in our path. With impoverished visual input, balance control and obstacle avoidance abilities become impaired due to misjudgment of distances and misinterpretation of spatial information^44^. The vestibulo-ocular reflex helps maintain visual fixation during head movements by generating compensatory short latency eye rotations. Vestibulospinal reflexes stabilize the head and help maintain upright stance, by triggering muscle activity in the neck, trunk and extremities^45^. Older people with vestibular hypofunction often have obvious impairments in posture and gait, which is characterized by postural instability and a broad-based, staggering gait pattern with unsteady turns^46^. Proprioception provides information to help coordinate each step and achieve the ideal foot placement through joint and muscle mechanoreceptors during walking, and quantitatively assessed impairments in tactile sensitivity at the ankle, vibration sense at the knee and knee joint position sense to be significant and independent risk factors for falls in a population of older people^47^. Muscle strength, drug use, oral health and environment also play an important role in falls in elderly^13^,^14^,^48^,^49^. TUGT, WS and GS are performance based tests. GS can partly test the function of musculoskeletal system^13^,^14^, while TUGT and WS are mobility and balance tests^9^,^11^ which can be affected by internal factors for falls such as age, body shape, vestibular sense, proprioception, muscle strength and vision^46^,^47^. Therefore, the influence of these internal factors of falls could be addressed by our tests. On the other hand, the influence of external factors of falls, for example, environment, can’t be presented by our tests. Also further researches should focus on how these factors affect the performance based tests and lead to falls, and the influence of external factors on falls.

It is still uncertain whether a single test can adequately predict adverse outcomes in high-functioning elderly persons. Specific diseases and impairments may affect certain aspects of function and may have an identical performance. Furthermore, using multiple measures increases reliability^17^, so some measurement accuracy may be gained by using multiple methods. Certain attempts have been made before to discover if using a combination of tests would more accurately predict falls^50^,^51^, yet the best study is currently inconclusive. In this way, our hypothesis is that the three most widely-used simple clinical physical tests—TUGT, GS and WS—would have stronger relationship with falls when combined. The final outcome confirmed this hypothesis. In addition, studying the combined use of tests helped us understand the role of WS in falls. According to our result, we suggest practioners develop and use some combination of these three tests as a screening tool for falls in older adults. Moreover, the prevention of falls should be accessed from multiple perspectives.

Our study has several limitations. First, this is a cross-sectional study, from which it is impossible to draw a definitive causal relationship. Therefore, investigation of the validity of the combination of the TUGT, GS and WS in predicting the risk of fall in older people using a prospective study design is recommended. Secondly, our study population is suburb-dwelling older adults in Hangu, Tianjin, China, which is narrow and not representative of all elderly.

In this cross sectional study, we found that the combination of better TUGT performance, greater GS and slower WS was correlated with the lowest possibility of falls in community-dwelling older adults. Further cohort research is needed to determine if the combination of these tests can serve as a simple but effective screening tool for fall risk. Moreover, the study result suggested the prevention of falls should be accessed from multiple perspectives.

## Additional Information

**How to cite this article**: Wang, X. *et al.* Mobility and Muscle Strength Together are More Strongly Correlated with Falls in Suburb-Dwelling Older Chinese. *Sci. Rep.* **6**, 25420; doi: 10.1038/srep25420 (2016).

## Figures and Tables

**Table 1:** Comparison of characteristics according to falls in past 12 months (only shows the statistically significant data).

Variables	Fall (n = 204)	Not fall (n = 888)
Sex, female, n (%)	135 (66.18)	474 (53.39)
Age (years)	68.91 ± 5.93	66.96 ± 5.92
Height (cm)	161.34 ± 9.16	163.24 ± 9.16
Cohabit with spouse, n (%)	152 (74.51)	720 (81.08)
Diabetes, n (%)	37 (18.10)	103 (11.60)
GDS degree, n (%)		
None	169 (82.76)	832 (93.67)
Slight depression	32 (15.76)	52 (5.88)
Moderate to severe depression	3 (1.48)	4 (0.45)
Muscle mass (kg/m^2^)	6.84 ± 1.03	7.07 ± 1.52

GDS: Geriatric Depression Scale.

Continuous variable are presented as mean ± SD, categorical variable are represented as number (%).

**Table 2:** Comparison of physical performance tests according to falls in past 12 months.

Variables	Fall (n = 204)	Not fall (n = 888)	P value
GS	0.35 ± 0.12	0.40 ± 0.12	<0.05
TUGT (s)	10.99 ± 4.62	9.65 ± 2.51	<0.05
WS (m/s)	0.95 ± 0.20	1.03 ± 0.20	<0.05

GS: grip strength/weight; TUGT: timed up and go test; WS: walking speed. Variable are presented as mean ± SD.

**Table 3:** Cutoff point of TUGT, GS and WS according to ROC curve.

Variable	Cutoff point	Sensibility	Specificity	AUC	95%CI	P value
TUGT	9.7150	0.578	0.577	0.609	0.566–0.652	<0.05
GS	0.3816	0.549	0.547	0.594	0.551–0.637	<0.05
WS	0.9963	0.561	0.566	0.586	0.541–0.632	<0.05

GS: grip strength/weight; TUGT: timed up and go test; WS: walking speed.

**Table 4:** Logistic regression analysis of the relationship between falls and TUGT, WS, GS and their combination.

Variables	Faller, n (%)	Crude		Model 1		Model 2	
		OR	95%CI	OR	95%CI	OR	95%CI
TUGT+	86 (14.38)	0.534*	0.392–0.726	0.609	0.444–0.837	0.622*	0.436–0.889
GS+	88 (15.25)	0.662*	0.487–0.900	0.971	0.673–1.400	1.063	0.703–1.607
WS+	88 (15.02)	0.598*	0.439–0.813	0.844	0.577–1.120	0.908	0.628–1.131
TUGT−, GS+	45 (18.99)	0.635*	0.416–0.971	0.945	0.587–1.521	0.940	0.554–1.595
TUGT+, GS−	43 (16.73)	0.506*	0.331–0.773	0.574*	0.372–0.885	0.563*	0.341–0.930
TUGT+, GS+	43 (15.61)	0.390*	0.256–0.593	0.623*	0.389–0.996	0.702	0.412–1.193
TUGT−, WS+	34 (17.62)	0.556*	0.355–0.871	0.725	0.455–1.155	0.858	0.519–1.419
TUGT+, WS−	32 (15.61)	0.481*	0.306–0.758	0.510*	0.322–0.808	0.555*	0.325–0.949
TUGT+, WS+	54 (13.74)	0.415*	0.283–0.608	0.573*	0.380–0.864	0.645	0.406–1.027
WS−, GS+	37 (17.62)	0.620*	0.400–0.961	0.747	0.475–1.174	0.794	0.475–1.327
WS+, GS−	37 (18.31)	0.712	0.458–1.107	0.956	0.593–1.539	0.956	0.554–1.649
WS+, GS+	51 (13.64)	0.483*	0.326–0.715	0.836	0.526–1.330	0.981	0.587–1.641
TUGT−, GS−, WS+	17 (26.98)	0.869	0.461–1.638	0.984	0.516–1.877	1.176	0.581–2.380
TUGT−, GS+, WS−	28 (25.93)	0.929	0.544–1.586	1.298	0.732–2.301	1.208	0.636–2.294
TUGT+, GS−, WS−	23 (20.91)	0.632	0.363–1.100	0.676	0.385–1.187	0.724	0.379–1.383
TUGT−, GS+, WS+	17 (13.18)	0.402*	0.221–0.733	0.690	0.732–2.301	0.779	0.384–1.577
TUGT−, GS−, WS+	20 (31.75)	0.396*	0.225–0.698	0.500	0.358–1.327	0.476*	0.238–0.952
TUGT+, GS+, WS−	9 (9.57)	0.291*	0.136–0.621	0.398*	0.279–0.897	0.394*	0.153–0.996
TUGT+, GS+, WS+	34 (13.88)	0.416*	0.257–0.672	0.747	0.433–1.290	0.867	0.472–1.594

GS: grip strength/weight; TUGT: timed up and go test; WS: walking speed.

Model 1adjusted age and sex.

Model 2 adjusted variables in Model 1 and height, cohabit with spouse, GDS, diabetes and muscle mass.

TUGT+: TUGT < 9.7150 s; TUGT−: TUGT > 9.7150 s; GS+: GS > 0.3816; GS−: GS < 0.3816; WS+: WS > 0.9973 m/s; WS−: WS < 0.9963 m/s

OR: odd ratio; CI: confidence Interval.

^*^means P < 0.05.

## References

[b1] Sattin R. W. Falls among older persons: a public health perspective. Annu Rev Public Health 13, 489–508, 10.1146/annurev.pu.13.050192.002421 (1992). 1599600

[b2] Tinetti M. E. & Williams C. S. Falls, injuries due to falls, and the risk of admission to a nursing home. N Engl J Med 337, 1279–1284, 10.1056/NEJM199710303371806 (1997). 9345078

[b3] Stevens J. A., Corso P. S., Finkelstein E. A. & Miller T. R. The costs of fatal and non-fatal falls among older adults. Inj Prev 12, 290–295, 10.1136/ip.2005.011015 (2006). 17018668 PMC2563445

[b4] Arnold C. M., Busch A. J., Schachter C. L., Harrison L. & Olszynski W. The relationship of intrinsic fall risk factors to a recent history of falling in older women with osteoporosis. J Orthop Sports Phys Ther 35, 452–460, 10.2519/jospt.2005.35.7.452 (2005). 16108586

[b5] Jiang C. Q. *et al.* [Effect of physical activity strength on the diabetes mellitus prevalence in the elderly under the influence of International Physical Activity Questionnaire]. Zhonghua Liu Xing Bing Xue Za Zhi 30, 462–465 (2009). 19799141

[b6] Guralnik J. M. *et al.* Lower extremity function and subsequent disability: consistency across studies, predictive models, and value of gait speed alone compared with the short physical performance battery. J Gerontol A Biol Sci Med Sci 55, M221–231 (2000). 10811152 10.1093/gerona/55.4.m221 PMC12149745

[b7] Cho S. I. & An D. H. Effects of a Fall Prevention Exercise Program on Muscle Strength and Balance of the Old-old Elderly. J Phys Ther Sci 26, 1771–1774, 10.1589/jpts.26.1771 (2014). 25435697 PMC4242952

[b8] Barry E., Galvin R., Keogh C., Horgan F. & Fahey T. Is the Timed Up and Go test a useful predictor of risk of falls in community dwelling older adults: a systematic review and meta- analysis. BMC Geriatr 14, 14, 10.1186/1471-2318-14-14 (2014). 24484314 PMC3924230

[b9] Shumway-Cook A., Brauer S. & Woollacott M. Predicting the probability for falls in community-dwelling older adults using the Timed Up & Go Test. Phys Ther 80, 896–903 (2000). 10960937

[b10] Dargent-Molina P. *et al.* Fall-related factors and risk of hip fracture: the EPIDOS prospective study. Lancet 348, 145–149 (1996). 8684153 10.1016/s0140-6736(96)01440-7

[b11] Verghese J., Holtzer R., Lipton R. B. & Wang C. Quantitative gait markers and incident fall risk in older adults. J Gerontol A Biol Sci Med Sci 64, 896–901, 10.1093/gerona/glp033 (2009). 19349593 PMC2709543

[b12] Kubicki A. Functional assessment in older adults: should we use timed up and go or gait speed test? Neurosci Lett 577, 89–94, 10.1016/j.neulet.2014.06.014 (2014). 24933540

[b13] Granacher U., Gollhofer A., Hortobagyi T., Kressig R. W. & Muehlbauer T. The importance of trunk muscle strength for balance, functional performance, and fall prevention in seniors: a systematic review. Sports Med 43, 627–641, 10.1007/s40279-013-0041-1 (2013). 23568373

[b14] Cheng Y. Y. *et al.* Can sit-to-stand lower limb muscle power predict fall status? Gait Posture 40, 403–407, 10.1016/j.gaitpost.2014.05.064 (2014). 24974126

[b15] Pijnappels M., van der Burg P. J., Reeves N. D. & van Dieen J. H. Identification of elderly fallers by muscle strength measures. Eur J Appl Physiol 102, 585–592, 10.1007/s00421-007-0613-6 (2008). 18071745 PMC2226001

[b16] Bongers K. T. *et al.* The predictive value of gait speed and maximum step length for falling in community-dwelling older persons. Age Ageing 44, 294–299, 10.1093/ageing/afu151 (2015). 25324333

[b17] Stewart A. L., Hays R. D. & Ware J. E. Jr. The MOS short-form general health survey. Reliability and validity in a patient population. Med Care 26, 724–735 (1988). 3393032 10.1097/00005650-198807000-00007

[b18] Quach L. *et al.* The nonlinear relationship between gait speed and falls: the Maintenance of Balance, Independent Living, Intellect, and Zest in the Elderly of Boston Study. J Am Geriatr Soc 59, 1069–1073, 10.1111/j.1532-5415.2011.03408.x (2011). 21649615 PMC3141220

[b19] Pavol M. J., Owings T. M., Foley K. T. & Grabiner M. D. Influence of lower extremity strength of healthy older adults on the outcome of an induced trip. J Am Geriatr Soc 50, 256–262 (2002). 12028206 10.1046/j.1532-5415.2002.50056.x

[b20] Liu N., Zeng L., Li Z. & Wang J. Health-related quality of life and long-term care needs among elderly individuals living alone: a cross-sectional study in rural areas of Shaanxi Province, China. BMC Public Health 13, 313, 10.1186/1471-2458-13-313 (2013). 23566211 PMC3642010

[b21] Li M. *et al.* Rural-urban differences in the long-term care of the disabled elderly in China. PLoS One 8, e79955, 10.1371/journal.pone.0079955 (2013). 24224025 PMC3818274

[b22] Silventoinen K., Magnusson P. K., Tynelius P., Kaprio J. & Rasmussen F. Heritability of body size and muscle strength in young adulthood: a study of one million Swedish men. Genet Epidemiol 32, 341–349, 10.1002/gepi.20308 (2008). 18271028

[b23] Miyatake N. *et al.* Linkage between oxygen uptake at ventilatory threshold and muscle strength in subjects with and without metabolic syndrome. Acta Med Okayama 61, 255–259 (2007). 17971842 10.18926/AMO/32895

[b24] Jurca R. *et al.* Association of muscular strength with incidence of metabolic syndrome in men. Med Sci Sports Exerc 37, 1849–1855 (2005). 16286852 10.1249/01.mss.0000175865.17614.74

[b25] Cruz-Jentoft A. J. *et al.* Sarcopenia: European consensus on definition and diagnosis: Report of the European Working Group on Sarcopenia in Older People. Age Ageing 39, 412–423, 10.1093/ageing/afq034 (2010). 20392703 PMC2886201

[b26] Bassett D. R. Jr. International physical activity questionnaire: 12-country reliability and validity. Med Sci Sports Exerc 35, 1396, 10.1249/01.MSS.0000078923.96621.1D (2003). 12900695

[b27] Chang N. T., Yang N. P. & Chou P. Incidence, risk factors and consequences of falling injuries among the community-dwelling elderly in Shihpai, Taiwan. Aging Clin Exp Res 22, 70–77, 10.3275/6627 (2010). 19934620

[b28] Yu P. L. *et al.* Prevalence and related factors of falls among the elderly in an urban community of Beijing. Biomed Environ Sci 22, 179–187, 10.1016/S0895-3988(09)60043-X (2009). 19725459

[b29] Lau E. M., Woo J. & Lam D. Neuromuscular impairment: a major cause of non-syncopal falls in elderly Chinese. Public Health 105, 369–372 (1991). 1754660 10.1016/s0033-3506(05)80596-7

[b30] Guideline for the prevention of falls in older persons. American Geriatrics Society, British Geriatrics Society, and American Academy of Orthopaedic Surgeons Panel on Falls Prevention. J Am Geriatr Soc 49, 664–672 (2001). 11380764

[b31] Lin M. R. *et al.* Psychometric comparisons of the timed up and go, one-leg stand, functional reach, and Tinetti balance measures in community-dwelling older people. J Am Geriatr Soc 52, 1343–1348, 10.1111/j.1532-5415.2004.52366.x (2004). 15271124

[b32] Morris R. *et al.* A comparison of different balance tests in the prediction of falls in older women with vertebral fractures: a cohort study. Age Ageing 36, 78–83, 10.1093/ageing/afl147 (2007). 17264139

[b33] Salarian A. *et al.* Analyzing 180 degrees turns using an inertial system reveals early signs of progression of Parkinson’s disease. Conf Proc IEEE Eng Med Biol Soc 2009, 224–227, 10.1109/IEMBS.2009.5333970 (2009). 19964471 PMC2954632

[b34] Zampieri C., S. A., Carlson-Kuhta P., Aminian K., Nutt J. G. & Horak F. B. An Instrumented Timed Up and Go Test Characterizes Gait and Postural Transitions in Untreated Parkinson’s Disease. J. Neurol. Neurosurg. Psychiatry 81, 171–176 (2010). 19726406 10.1136/jnnp.2009.173740 PMC3065923

[b35] Chu L. W., Chi I. & Chiu A. Y. Incidence and predictors of falls in the chinese elderly. Ann Acad Med Singapore 34, 60–72 (2005). 15726221

[b36] Oh D. H. *et al.* Intensive exercise reduces the fear of additional falls in elderly people: findings from the Korea falls prevention study. Korean J Intern Med 27, 417–425, 10.3904/kjim.2012.27.4.417 (2012). 23269883 PMC3529241

[b37] Ballard J. E., McFarland C., Wallace L. S., Holiday D. B. & Roberson G. The effect of 15 weeks of exercise on balance, leg strength, and reduction in falls in 40 women aged 65 to 89 years. J Am Med Womens Assoc 59, 255–261 (2004). 16845754

[b38] Freiberger E., Haberle L., Spirduso W. W. & Zijlstra G. A. Long-term effects of three multicomponent exercise interventions on physical performance and fall-related psychological outcomes in community-dwelling older adults: a randomized controlled trial. J Am Geriatr Soc 60, 437–446, 10.1111/j.1532-5415.2011.03859.x (2012). 22324753

[b39] Orr R., Raymond J. & Fiatarone Singh M. Efficacy of progressive resistance training on balance performance in older adults: a systematic review of randomized controlled trials. Sports Med 38, 317–343 (2008). 18348591 10.2165/00007256-200838040-00004

[b40] Stone E., Skubic M., Rantz M., Abbott C. & Miller S. Average in-home gait speed: investigation of a new metric for mobility and fall risk assessment of elders. Gait Posture 41, 57–62, 10.1016/j.gaitpost.2014.08.019 (2015). 25245308

[b41] Tsang W. W. *et al.* The effects of aging on postural control and selective attention when stepping down while performing a concurrent auditory response task. Eur J Appl Physiol 113, 3021–3026, 10.1007/s00421-013-2740-6 (2013). 24091870

[b42] Bunday K. L. & Bronstein A. M. Visuo-vestibular influences on the moving platform locomotor aftereffect. J Neurophysiol 99, 1354–1365, 10.1152/jn.01214.2007 (2008). 18184886

[b43] Mitchell R. J., Lord S. R., Harvey L. A. & Close J. C. Associations between obesity and overweight and fall risk, health status and quality of life in older people. Aust N Z J Public Health 38, 13–18, 10.1111/1753-6405.12152 (2014). 24494939

[b44] Lord S. R. Visual risk factors for falls in older people. Age Ageing 35 Suppl 2, ii42–ii45, 10.1093/ageing/afl085 (2006). 16926203

[b45] Sturnieks D. L., St George R. & Lord S. R. Balance disorders in the elderly. Neurophysiol Clin 38, 467–478, 10.1016/j.neucli.2008.09.001 (2008). 19026966

[b46] Tian J. R., Crane B. T., Wiest G. & Demer J. L. Effect of aging on the human initial interaural linear vestibulo-ocular reflex. Exp Brain Res 145, 142–149, 10.1007/s00221-002-1111-z (2002). 12110953

[b47] Lord S. R., McLean D. & Stathers G. Physiological factors associated with injurious falls in older people living in the community. Gerontology 38, 338–346 (1992). 1473733 10.1159/000213351

[b48] Pit S. W. *et al.* A Quality Use of Medicines program for general practitioners and older people: a cluster randomised controlled trial. Med J Aust 187, 23–30 (2007). 17605699 10.5694/j.1326-5377.2007.tb01110.x

[b49] Maruya M. S. & Ohnuma T. The effect of wearing denture and changes of occlusal position on body sway in edentulous patient. Jpn Prosthodont Soc 44, 781–785 (2000).

[b50] Boulgarides L. K., McGinty S. M., Willett J. A. & Barnes C. W. Use of clinical and impairment-based tests to predict falls by community-dwelling older adults. Phys Ther 83, 328–339 (2003). 12665404

[b51] Makizako H. *et al.* The combined status of physical performance and depressive symptoms is strongly associated with a history of falling in community-dwelling elderly: cross-sectional findings from the Obu Study of Health Promotion for the Elderly (OSHPE). Arch Gerontol Geriatr 58, 327–331, 10.1016/j.archger.2014.01.001 (2014). 24525136

